# Impacts of studying in a regional medical campus on practice location

**Published:** 2018-03-27

**Authors:** Anouk Utzschneider, Michel Landry

**Affiliations:** 1 Université de Sherbrooke, Quebec, Canada; 2 Centre de formation médicale du Nouveau-Brunswick, Moncton, New Brunswick, Canada

## Abstract

**Background:**

New Brunswick, a bilingual Canadian province without a medical school, negotiated an agreement in 1967 in which places were reserved for francophone medical students in the province of Quebec. In 2006, the Centre de Formation Médicale du Nouveau-Brunswick (CFMNB), a regional medical campus (RMC) of Université de Sherbrooke for its provincial francophone medical students, was established to increase the likelihood of graduates setting up practice in the region. Practice locations of the initial 5 cohorts of CFMNB were analysed to compare data with francophone students trained in Quebec.

**Methods:**

Practice locations were determined through Scott’s Medical Database and provincial public registries. Chi-square and relative risk probability were used to examine the relationship between training location and practice location.

**Results:**

Doctors trained at CFMNB were 1.4 times more likely to be practicing in Atlantic Canada compared to those trained at Université de Sherbrooke (main campus) before 2006. Those trained at CFMNB were 1.3 times more likely to go on to practice in the region compared to those trained at Université Laval or Université de Montréal.

**Conclusion:**

This study supports the hypothesis that individuals completing a medical program in a Francophone RMC in New Brunswick increases the likelihood of them later practicing in the province or in the wider Atlantic Canada region.

## Abstract (fr)

**Contexte:**

Le Nouveau-Brunswick, une province canadienne bilingue ne possédant pas de Faculté de médecine, a négocié en 1967 une entente avec le Québec afin de réserver des places pour ses étudiants francophones souhaitant étudier la médecine. En 2006, le Centre de formation médicale du Nouveau-Brunswick (CFMNB), un campus médical régional (CMR) de l’Université de Sherbrooke, a été créé afin de permettre aux Néo-Brunswickois d’étudier la médecine en français dans leur province. L’un des objectifs principaux du CFMNB était d’augmenter les probabilités que les diplômés en médecine s’établissent dans la région. Les lieux de pratique des médecins issus des 5 premières cohortes du CFMNB ont été analysés afin de les comparer avec ceux des médecins francophones d’origine néo-brunswickoise formés au Québec.

**Méthodes:**

Les lieux de pratique ont été déterminés via le répertoire des médecins Scott’s et les registres publics provinciaux. Des tests de Chi carré et des analyses de risque ont été conduits afin d’étudier la relation entre le lieu de formation et le lieu de pratique.

**Résultats:**

Les médecins formés au CFMNB étaient 1.4 fois plus enclins à travailler au Canada atlantique comparativement à ceux formés au campus principal de l’Université de Sherbrooke avant 2006. Les médecins formés au CFMNB étaient également 1.3 fois plus enclins à travailler dans la région que ceux formés à l’Université Laval ou à l’Université de Montréal.

**Conclusion:**

Cette étude supporte l’hypothèse selon laquelle le fait de compléter un programme de médecine francophone dans un CMR francophone au Nouveau-Brunswick augmente les chances de pratiquer au Nouveau-Brunswick ou dans la grande région du Canada atlantique.

## Introduction

Medical doctor shortage and maldistribution is a constraint to equitable delivery of healthcare in most countries^1-4^ and the need for more doctors tends to be greater in rural regions, typically at some distance from most medical schools.^5-7^ Many studies suggest that medical students who are exposed to a certain region during medical training are more likely to establish a medical practice in their training region than students who do not get this exposure.^1^,^8^,^9^ This is reflected in the growth of Regional Medical Campuses (RMCs) and the expansion of medical education training programs in local communities in many countries.^10^

In New Brunswick, a geographically small and sparsely populated Canadian province (population of 753,900), access to quality health care has been an issue for rural communities and language minorities as the province is predominantly rural (48%) and has a large French-speaking minority (approximately 33% of the population). Moreover, the French population has traditionally settled in rural communities and is generally poorer and older than the province’s English majority.^11^ Thus, New Brunswick’s French minority identifies as an underserved, vulnerable group lacking sufficient services access to services in its own language.^12^

Since New Brunswick is one of two Canadian provinces without a medical school, it negotiated an agreement in 1967 that reserved places for New Brunswick students in three French medical schools in Quebec (Université Laval, Université de Montréal and Université de Sherbrooke). In order to qualify for a reserved space, applicants must have had a permanent address in New Brunswick for the last twelve months prior to applying. The distance between the Quebec medical schools and most francophone communities in New Brunswick ranges between 700 km and 1,000 km.^1^ In 1981, a clinical teaching program was established at Moncton’s Dr. Georges L Dumont Regional Hospital, New Brunswick’s largest francophone hospital. Since 1999, the entire 24 months of the Family Medicine Program have been offered in New Brunswick through the Université de Sherbrooke. In 2006, Centre de Formation Medicale du Nouveau-Brunswick (CFMNB), a regional medical campus (RMC) of Université de Sherbrooke, was established in Moncton, New Brunswick (the province’s most populated city; 72,000 in 2016;), as the first cross-provincial RMC in Canada. As a result, New Brunswickers can now study medicine in French in their home province. A limited number of places are still reserved each year for francophone New Brunswickers at Université Laval and Université de Montréal, but all New Brunswickers studying at Université de Sherbrooke are located at CFMNB. The CFMNB was developed under the hypothesis that completing a medical program in the province would increase the likelihood of graduates to practice in New Brunswick or in the general Atlantic region (which includes New Brunswick, Prince Edward Island, Nova Scotia, and Newfoundland & Labrador).

The Atlantic region is predominantly Anglophone but includes numerous pockets of underserved Francophone communities.

This paper presents early evidence of the impact on further practice location of the first cross-provincial regional medical campus in Canada that is offered as a fully distributed medical program. Data from the initial five cohorts of the CFMNB were analysed to show how they compared with other Francophone students who trained outside of New Brunswick.

## Methods

### Study populations

The study targeted three different populations: 1) participants who were admitted to study medicine at Université de Sherbrooke through the Quebec-New Brunswick agreement between 1973 and 2005; 2) participants who graduated from Université de Sherbrooke’s medical program in New Brunswick between 2010 and 2014 (admitted between 2006 and 2010); and 3) participants who were admitted at Université Laval and Université de Montréal through the Quebec-New Brunswick agreement between 1973 and 2010. Université de Sherbrooke has offered clinical teaching opportunities for the past 35 years in New Brunswick and a Family Medicine Program there for the past 17 years.

### Participants who trained through the Quebec-New Brunswick agreement

Data about practice location of participants trained through the Quebec-New Brunswick agreement between 1973 and 2000 were collected as part of an earlier study conducted between October 2007 and July 2008. The focus of the 2007-2008 study was on the relationship between recruitment and retention of doctors and exposure to the province during medical training.^1^ Information on practice location was obtained through the support of the Maritime Provinces Higher Education Commission (MPHEC), which is responsible for funding the reserved spots in Quebec used by New Brunswickers as part of the interprovincial agreement.

The names of participants who were admitted through the Quebec-New Brunswick agreement from 2001 to 2010 were also obtained from MPHEC. Practice locations were identified using Scott’s Medical Database, which lists those physicians in Canada who are members of the Canadian Medical Association and permit release of their information.^13^ Some additional data were also retrieved from provincial public registries across Canada.

### Université de Sherbrooke RMC and CFMNB trainees

Names of participants who graduated from CFMNB between 2010 and 2014 were obtained from an internal administrative database. Practice locations were identified using Scott’s Medical Database. Some additional data were also retrieved from provincial public registries across Canada. Doctors identified as working on locums were excluded from the study because of the temporary nature of their work location.

Family residency programmes are two years in duration in Canada and therefore all participants from a family residency programme would potentially have been practicing at the time of follow ups. Specialty residency programmes, however have a minimum duration of four years. Specialists’ practice locations are therefore missing for graduating cohorts of 2013 and 2014 in this study.

Some students who were admitted through the agreement may have never graduated or may have chosen another career after graduating from medical school, but information on this group was unavailable. This group of students is therefore part of the “unsuccessfully located group.” However, students who did not graduate from the CFMNB were excluded from the study since this information was available. This is why we considered “graduates” for CFMNB and “admitted” for Université de Sherbrooke, Université Laval and Université de Montréal for this study.

The study received ethical approval from Université de Moncton’s Institutional Research Ethics Review Board.

### Data analysis

Practice locations of the three groups were compared according to the classification Atlantic Canada (New Brunswick, Prince Edward Island, Nova Scotia and Newfoundland & Labrador) vs Outside Atlantic Canada (all other province and other countries). Comparisons were also made regarding whether students had followed a family medicine or specialty career path. Chi-square and relative risk (RR) probability tests were conducted to examine the relationship between training location and practice location. The association between training location and practice location was determined using Cramer’s 𝜑_2_ from the chi-squared test. All analyses were conducted using IBM SPSS Statistics 24.

## Results

Of the 251 potential participants admitted from 1973 to 2005 at Université de Sherbrooke on our list, 208 were identified from the available data (see Table 1). Median year of admission was 2000 and 59% of this group were women.

**Table 1:** Characteristics of study participants

	All participants (n= 457)	Family doctors (n= 283)	Specialists (n=174)
	n (%)	n (%)	n (%)
Total number of graduates practicing in Atlantic Canada from the three Francophone programs at time of tracking	311 (68%)	220 (78%)	91 (52%)
***Study population (by school)***			
Université de Sherbrooke main campus (before 2006)	208 (46%)	128 (45%)	80 (46%)
Université Laval	122 (27%)	64 (23%)	58 (33%)
Université de Montréal	61 (13%)	40 (14%)	21 (12%)
Centre de Formation Médicale du Nouveau-Brunswick (after 2006)	66 (14%)	51 (18%)	15 (9%)
Sex, female	225 (49%)	133 (47%)	92 (53%)

Of the 218 potential participants admitted from 1973 to 2010 at Université Laval and Université de Montréal on our list, 183 were successfully located. Median year of admission was 2002 and 56% were women.

We were able to locate all 66 doctors who were admitted from 2006 to 2010 at CFMNB. Women accounted for 68% of this group. Women globally represented 62% of all admissions to the CFMNB, but those students who entered a specialty residency program in 2013 or 2014 (the majority of whom were males) were not yet practicing at the time of the study and were therefore not represented in this group. We found no significant differences in terms of practice locations between the five CFMNB cohorts when comparing *Atlantic Canada* vs *Outside Atlantic Canada*.

There were 457 participants across the three groups.

### Practice location of doctors trained at Université de Sherbrooke’s main campus (before 2006) compared to those trained at CFMNB (after 2006)

According to our results, 86% (n = 57 out of 66) of doctors trained at CFMNB and 63% (n = 132 out of 208) of doctors trained at Université de Sherbrooke (main campus) were practicing in Atlantic Canada (*X*_2_ = 12.28, p < .001) at the time of the study. The association between training location and practice location was moderate (Cramer’s 𝜑_2_ =0.21). Those who were trained at CFMNB had 1.36 times the likelihood (95% CI = 1.18-1.57) of practicing in Atlantic compared to those trained at Université de Sherbrooke.

In terms of career path, 88% (n = 45 out of 51) of family doctors trained at CFMNB and 78% (n = 100 out of 128) of those trained at Université de Sherbrooke were practicing in Atlantic Canada (*X*_2_= 2.42, p > 0.05). The association between practice location and training location was small (Cramer’s 𝜑_2_ =0.12) and doctors trained at CFMNB were 1.12 times more likely (95% CI = 0.99-1.29) to be practicing in Atlantic Canada compared to those who trained at Université de Sherbrooke.

We found that 80% (n = 12 out of 15) of doctors trained at CFMNB and 40% (n = 32 out of 80) of those trained at Université de Sherbrooke who followed a specialist career path set up practice in Atlantic Canada (*X*_2_ = 8.13, p < 0.05). The association between training location and practice location for specialists only was moderate (Cramer’s 𝜑_2_ =0.29). According to the relative risk analysis, specialists trained at CFMNB were twice as likely (95% CI = 1.38-2.89) to be practicing in the Atlantic region compared to those trained at Université de Sherbrooke.

### Practice locations of CFMNB, Université Laval and Université de Montréal graduates

When comparing graduates from CFMNB with those from Université Laval and Université de Montréal together, we find that 86% (n = 57 out of 66) of doctors trained at CFMNB and 67% (n = 123 out of 183) of those trained at Université Laval or Université de Montréal were practicing in Atlantic Canada (*X*_2_ = 8.88, p < 0.05). The association between training location and practice location was small (Cramer’s 𝜑_2_ =0.19). Those who were trained at CFMNB were 1.29 times (95% CI = 1.12-1.48) more likely to be practicing in Atlantic Canada compared to those trained at Université Laval or Université de Montréal.

For family doctors only, 88% (n = 45 out of 51) of doctors trained at CFMNB and 73% (n = 76 out of 104) of those trained at Université Laval and Université de Montréal are practicing in Atlantic Canada (*X*_2_ = 4.59, p < 0.05). The association between training location and practice location was small (Cramer’s 𝜑_2_ =0.17). Those who were trained at CFMNB were 1.21 times (95% CI = 1.04-1.41) more likely to be practicing in Atlantic Canada compared to those trained at Université Laval or Université de Montréal.

In terms of specialists, 80% (n = 12 out of 15) of doctors trained at CFMNB and 62% (n = 49 out of 79) of those trained at Université Laval or Université de Montréal were practicing in Atlantic Canada (*X*_2_= 2.27, p > 0.05). The association between practice location and training location was small (Cramer’s 𝜑_2_ =0.16). According to the relative risk analysis, specialists trained at CFMNB were 1.35 times (95% CI = 0.99 – 1.84) more likely to be practicing in the Atlantic region compared to those trained at Université Laval or Université de Montréal.

## Discussion

The CFMNB was established in Moncton in 2006 as the first cross-provincial RMC in Canada with the objective of training doctors locally, in French, as a way of increasing the likelihood of graduates going on to set up practice in New Brunswick or the wider Atlantic region. This may be the first study to compare the practice locations of doctors trained within the same program (Université de Sherbrooke) but in different provinces. Comparing practice locations of students from the two other francophone universities who reserve places for New Brunswickers allows for a deeper understanding of the subject.

We found that doctors trained at CFMNB were 1.4 times more likely to practice in Atlantic than those trained at Université de Sherbrooke’s main campus. This result is consistent with reports in the literature, which state that medical students who are exposed to a certain geographic region during medical training are more likely to establish a medical practice in the training region than other students.^1,8,9^ When looking at specialists only, we found significant results showing that specialists trained at CFMNB were twice as likely to practice in the Atlantic region than those trained in Sherbrooke. Since the first 15 specialists trained initially at CFMNB entered the workforce in 2015 (12 of whom entered the workforce in New Brunswick), it is possible that they took advantage of the vacant positions in the region, resulting in a substantial rise in the numbers. We expect that the number of CFMNB-trained specialists setting up practice in Atlantic Canada may decrease in the years to come due to fewer practice opportunities. However, these early results indicate that many CFMNB graduates are establishing their practices in Atlantic Canada. For family doctors, since Université de Sherbrooke’s residency programme in family medicine has been offered in full in New Brunswick since 1999 (and partially since 1981), the difference in practice locations between doctors trained at undergraduate level at the main site in Sherbrooke and those trained at CFMNB are less striking.

We found that those trained at CFMNB were 1.3 times more likely to practice in Atlantic Canada than those trained at Université Laval or Université de Montréal, but the association between training location and practice location was not as strong as it was when comparing with Université de Sherbrooke. In this case we found a significant difference for family doctors but not for specialists.

In regards to family doctors and specialists from New Brunswick who do not practice in the Atlantic region, the vast majority of them have set up practice in Quebec (78% of doctors trained at CFMNB who practice outside the Atlantic region are in Quebec, compared to 87% for those trained at Université de Sherbrooke, and 90% for those trained at Université de Montréal or Université Laval). Ontario is the second most popular destination for these graduates but it comes far behind Quebec. We found very few Francophone doctors from New Brunswick practicing in other parts of Canada. We were unable to find data to track expatriated Francophone doctors from New Brunswick. Therefore we do not know exactly how many doctors belong to this group.

To our knowledge, there are no specific incentives in terms of salary or job security that would draw new doctors to New Brunswick or the Atlantic region more than to other regions of the country. Additionally, according to the information collected by CAPER^14^ in 2016, physician opportunities (for family doctors and specialists) are not higher in the Atlantic region than elsewhere in Canada.

The linguistic component could be an important factor influencing the doctors who were trained in francophone universities to practice in francophone settings. This tendency could explain higher concentrations of doctors trained through the Quebec – New Brunswick agreement to practice in Atlantic Canada, Quebec and Ontario. Data from the Canadian Post-MD Education Registry (CAPER)^15^ indicates that approximate 90% of all doctors who were awarded an M.D. Degree from a francophone university in 2014 were practicing in Quebec two years after exiting training. In comparison, only around 1% of all doctors who completed their M.D. Degree at Memorial and Dalhousie universities (both located in the Atlantic region) in 2014 practiced in Quebec two years after exiting training. Half of them stayed in the Atlantic region and others went to predominantly Anglophone Canadian provinces. We believe that students’ attachment to the region and the opportunity to work in a francophone or bilingual environment influences their choice of practice location.

A limitation of this study was that it did not take into account exposure to the region during undergraduate training rotations and postgraduate training. Opportunities for rotations and residency in the province have evolved over time and may have influenced our results. Job availability and security have also evolved over time in different ways in each province, and various policies to encourage or discourage doctors from establishing their practice in certain regions have also been pursued. This historical aspect is difficult to capture retrospectively but represents a potential confounder. Moreover, this study relied on administrative data, which did not capture a wide range of factors that may have affected decision making about work locations, such as a spouse’s ability to work in the region^16^. The identification of practice location of participants at a single point in time is another limitation of the study. Consequently, it is unclear whether any of the doctors tracked from Université de Sherbrooke, Université Laval and Université de Montréal had moved their practices within or outside of the Atlantic region before or after the information was collected for this study.

### Conclusion

This study supports the hypothesis that completing a medical program in a francophone RMC in New Brunswick increases the likelihood of a graduate later practicing in the province or in the wider Atlantic Canada region. Our results suggest that doctors who have completed their ungraduated training in a cross provincial campus tend to practice in the region in which they were trained. However, results vary between family doctors and specialists. Results about practice location must also be interpreted in regard to the availability of positions, as practice locations are not solely defined by a doctors’ desires.

The success of CFMNB is of great significance for New Brunswick’s francophone population. The CFMNB not only contributes to the recruitment of doctors, it also trains doctors who are better prepared to face New Brunswick’s linguistic and geographic realities. Further research is required to gain better insight into long-term impacts of regional medical campuses and how they respond to the needs of specific regions.
